# Use of antibiotic prophylaxis in conjunction with dental implant surgery in Sweden- A cross-sectional study

**DOI:** 10.1186/s12903-025-06579-x

**Published:** 2025-07-25

**Authors:** Palwasha Momand, Aron Naimi-Akbar, Nina Hämén von Essen, Bengt Götrick

**Affiliations:** 1 Department of Oral Diagnostics, Faculty of Odontology, Malmö University, Malmö, Sweden; 2 Health Technology Assessment-Odontology (HTA-O), Faculty of Odontology, Malmö University, Malmö, Sweden; 3 Smile Dentistry, Malmö, Sweden; 4 Public Dental Service Stockholm, Stockholm, Sweden; 5 Department of Orofacial Medicine, Faculty of Odontology, Malmö University, Malmö, Sweden

**Keywords:** Cross-sectional study, Antibiotic prophylaxis, Dental implant surgery, Antibiotic resistance

## Abstract

**Background:**

Antibiotic prophylaxis in dental implant surgery remains a contentious topic, with varying guidelines and clinical practices worldwide.

**Aim:**

Report how Swedish dentists use antibiotic prophylaxis during dental implant surgery, based in a review of patient records.

**Method:**

This retrospective, cross-sectional study evaluated the antibiotic prophylaxis habits of Swedish dentists, focusing on the relationship between surgical complexity and antibiotic use.

**Results:**

Data from 450 patient records with registered implant surgeries at two major dental care providers, one public and one private, were analysed. Thirty-seven clinics provided the data, and 109 dentists performed the surgeries. Findings revealed that 72.2% of implant surgeries were straightforward procedures, with no administration of antibiotic prophylaxis in 70.5% of these. Conversely, 90% of bone augmentation cases involved antibiotic use, particularly in complex protocols utilizing synthetic materials and membranes. Overall, 54.4% of patients in the study population received no antibiotic prophylaxis at all. Surgical complexity was a significant predictor for antibiotic administration, while patient-specific factors, such as age, chronic diseases, and tobacco use, had limited influence.

**Conclusion:**

Restrained antibiotic use in implant surgery in Sweden reflects alignment with current stewardship goals, particularly in straight forward procedures. However, continued administration in some uncomplicated cases and frequent use in complex surgeries highlight the need for clearer, evidence-based guidelines and standardized antibiotic protocols.

## Introduction

Antibiotic resistance has emerged as one of the most significant global health threats of the 21st century [1]. The World Health Organization (WHO) has repeatedly warned that the overuse and misuse of antibiotics in both human and veterinary medicine are rapidly eroding the effectiveness of many types of antibiotics [2, 3]. The rise of antibiotic resistance not only threatens the ability to treat ongoing infections, it also reduces the ability to use antibiotics to prevent infections, a cornerstone of modern medical care [4]. Resistant bacteria make it increasingly difficult to manage infections, leading to potential complications, prolonged hospital stays, and higher mortality. These consequences of antibiotic resistance are particularly concerning in the context of surgical procedures, where the risk of postoperative infection is a primary concern [5].

In the field of dentistry, antibiotics are used to either treat existing infections or prophylactically to prevent infections in conjunction with specific treatments. The purpose of antibiotic prophylaxis in dental implant surgery is to reduce the risk of infection which could lead to complications such as implant loss [6]. However, as the global crisis of antibiotic resistance intensifies, the use of prophylactic antibiotic in dentistry has become under increasing scrutiny. While there is a longstanding tradition of prescribing antibiotics in conjunction with dental implant surgery [7], the efficacy and necessity of this practice remains uncertain [8–11]. The benefits of antibiotics prophylaxis in dental implant surgery are debated, with some evidence suggesting that the small risk of infection that could potentially lead to implant loss may not justify routine antibiotic use, particularly in healthy patients [8, 11, 12].

Moreover, conflicting clinical guidelines and a lack of universal consensus within the dental community further complicate decision-making. The 4th European Association for Osseointegration (EAO) Consensus Conference (2015) [13] recommend no prophylaxis for straightforward implant surgeries. In contrast to this, the Recommendations from the Spanish Society of Implants (2022) [14], support antibiotic prophylaxis for all implant procedures, and the Consensus Report by the Italian Academy of Osseointegration (2021) [15] advocate antibiotics even for straightforward cases. In Sweden, The Swedish Medical Product Agency conformed a national guideline in 2012 concluding that there was limited evidence for the benefit of antibiotic prophylaxis and suggesting that prophylaxis may be considered in certain cases, e.g. in a patient with a combination of several risk factors [16]. However, the guideline does not clarify which risk factors are referred to.

While randomized controlled trials (RCTs) that investigate the effect of antibiotic prophylaxis in conjunction with implant surgery exist, most focus on straightforward implant surgeries [17–21], leaving a gap in evidence for complex procedures like simultaneous bone augmentation. Furthermore, survey-based studies have revealed a high frequency of antibiotic prescribing among dental clinicians, but these studies are often limited by sparse response rates (2–40%) and lack of clarity on procedural contexts [22–26]. In some of the survey studies, it remains unclear whether reported high antibiotic use reflects complex procedures or universal prophylaxis regardless of case difficulty [22, 25].

These uncertainties are compounded by the broader issue of antibiotic resistance: unnecessary use of antibiotics in dental procedures contributes to the overall burden of resistance, potentially undermining the effectiveness of these drugs for future generations. As the global health community strives to combat antibiotic resistance, there is an increasing call for more judicious and evidence-based use of antibiotics across all medical fields, including dentistry [4].

Our hypothesis is that when it comes to antibiotic prophylaxis in conjunction with implant surgery, dentists in Sweden are more likely to use antibiotics in complex surgeries compared to straightforward. The aim of this study is to obtain a report on how dentists in Sweden use antibiotic prophylaxis in conjunction with dental implant surgery based on a review of patient records.

## Materials and methods

### Study design

This retrospective, cross-sectional study examined antibiotic prophylaxis practices in dental implant surgery in Sweden. The Swedish Ethical Review Authority approved this study (daybook no. [Dnr]: 2023-07228-01), which adhered to the Strengthening the Reporting of Observational Studies in Epidemiology (STROBE) guidelines for reporting observational studies [27]. The study was carried out with complete anonymity at both the patient and the dentist level.

### Study population and data collection

This study retrieved 450 patient records: 250 from the Public Dental Service (PDS) Stockholm and 200 from Smile Dentistry, a private dental service organization. The number of patient records from the PDS Stockholm was proportionately higher than those from Smile Dentistry due to the larger size and scope of the PDS Stockholm, which operates more than 80 clinics across one of Sweden’s largest counties compared to Smile Dentistry’s 65 clinics nationwide. The patient records were selected based on registry codes for implant surgery. Consecutive records of implant surgery were retrieved from 1 January 2023 and going backwards in time until the predetermined number of records was collected. The target records were extracted from the electronic patient journal databases of each organization. To ensure comprehensive data collection, we decided to include information from 1 month before and 1 month after the implant procedure. The inclusion criterion was that the dentist was an oral and maxillofacial surgeon, a periodontist, or a general dentist who routinely performed implant procedures, defined as 50 or more dental implant surgeries each year. Thirty-seven clinics provided the data, and 109 dentists performed the surgeries.

Extracted data included:

- *Demographics*: Age, gender.
- *Health factors*: General health conditions, medication use, and tobacco use.
- *Procedural details*: Type of implant surgery (e.g., straightforward, bone augmentation, sinus lift, one-step and two-step implant placement), number of implants, and position.
- *Antibiotic prophylaxis*: Type, dosage, and duration.
- *Postoperative recommendations*: Chlorhexidine rinse.

The definition of types of surgeries were the following:

- Straightforward surgeries: single implant placements in generally healthy individuals with adequate bone volume and soft tissue conditions, not needing bone augmentation.
- Complex surgeries: sinus lifts, bone augmentation (minor and major), and multi-implant placements.

### Statistical analysis

The sample size calculation was based on expected antibiotic administration rates of 75% for straightforward surgeries and 90% for complex procedures. With a Type I error rate of 0.05 and 80% power, a minimum of 100 patients per group was required. This ensured adequate statistical power to detect significant differences between the straightforward group and the complex procedures group.

The statistical analysis for this study was designed to comprehensively evaluate the use of antibiotic prophylaxis in conjunction with implant surgeries. The analyses focused on describing the data, identifying patterns, and assessing relationships between variables to draw meaningful conclusions about clinical practices.

Descriptive analysis was performed to summarize the characteristics of the study population and key variables. Frequencies and percentages were presented to describe categorical data, such as the type of implant surgery and the proportion of patients receiving antibiotic prophylaxis.

Logistic regression analysis was used to explore the factors associated with the administration of antibiotic prophylaxis. The outcome variable was a binary indicator of whether antibiotics were prescribed (Yes/No). Independent variables included type of surgery (straightforward, sinus lift, bone augmentation) and patient-specific factors (e.g., tobacco use, health conditions, age). The results were reported as odds ratios (OR) with 95% confidence intervals, quantifying the likelihood of antibiotic administration for different subgroups. This method accounted for multiple covariates simultaneously, offering insights into the predictors of antibiotic use.

Statistical analyses were performed using the Statistical Package for the Social Sciences (SPSS, version 27.0; IBM SPSS Statistics for Windows, IBM Corp., released in 2020).

## Results

### Patient characteristics

Table 1 presents patient characteristics and an overview of which patients received antibiotic prophylaxis and which did not.

**Table 1:** Patient data characteristics and antibiotic prophylaxis

Characteristics	Total	Antibiotics	
	*n*	Yes %	No %
**Sex**			
Male	216	47.7	52.3
Female	234	43.6	56.4
*Total*	*450*	*45.6*	*54.4*
**Age groups**			
20–44 years	57	33.3	66.7
45–59 years	184	41.3	58.7
60–74 years	137	48.9	51.1
75 + years	72	59.7	40.3
**Tobacco use**			
None	354	42.4	57.6
Cigarettes	23	43.5	56.5
Snuff	28	28.6	71.4
**Health conditions**			
Diabetes	29	58.6	41.4
Cancer	13	44.3	53.8
Osteoporosis	6	100.0	0.0
**Type of surgery**			
Straightforward	325	29.5	70.5
Closed sinus lift without bone augmentation	40	85.0	15.0
Immediate placement without bone augmentation	7	71.4	28.6
Bone augmentation	35	82.9	17.1
Closed sinus lift with bone augmentation	22	95.5	4.5
Immediate placement with bone augmentation	13	92.3	7.7
Others*	8	100.0	0.0
**Number of implants**			
Single implant	322	43.2	56.8
2–3 implants	119	51.3	48.7
4–6 implants	8	62.5	37.5

*Others included patients undergoing extraction of ajacent tooth during implant placement (7 patients), or sinus life without stimoultanous bone augmentation(1 patient))

The gender distribution in the study population was 48.0% male (*n* = 216) and 52.0% female (*n* = 234) patients. Mean participant age was 59.9 years (SD = 13.4, range 20–94). Patients aged 45–59 years constituted the largest group (40.9%), followed by those aged 60–74 (30.4%), 75+ (16.0%), and 20–44 (12.7%). Tobacco use was reported by 12.6% of patients, with 5.7% using cigarettes, and 6.9% using snuff. Data on tobacco use was missing for 45 (10.0%) of the patients. The health conditions of the study population included a smaller proportion of individuals with systemic conditions such as diabetes (6.4%), cancer (2.9%), and osteoporosis (1.3%), Table 1.

### Procedural details

Most procedures were straightforward implant placements (*n* = 325 patients; 72.2%). Other types of surgeries that did not involve bone augmentation were closed sinus lift (*n* = 40; 8.9%), immediate implant placement (*n* = 7; 1.6%), and others (*n* = 8; 1.8%), Table 1. Closed sinus lift, or a intra-crestal lift, was defined as a technique in which the sinus membrane was elevated through the implant osteotomy site without creating a lateral window. The group labelled as ‘Others’ consisted of patients that underwent an extraction of an adjacent tooth during implant placement (7 patients), or an open sinus lift without simultaneous bone augmentation (1 patient). Open sinus lift (lateral window sinus lift) involved creating a lateral access point in the maxillary sinus.

Bone augmentation was performed in 70 patients (15.6%). Of these solely bone augmentation was performed in 35 patients (7.8%); bone augmentation in conjunction with closed sinus lift in 22 patients (4.9%); and direct installation with bone augmentation in 13 patients (2.9%), Table 1.

The study population consisted of 450 patients who received a total of 616 implants. Most patients received implants from Straumann^®^: (*n* = 411 patients; 91.7%), followed by Nobel^®^ (*n* = 25; 5.6%), Brånemark^®^ (*n* = 7; 1.6%); Neosspro Active^®^ (*n* = 3; 0.7%); and Astra^®^ (*n* = 2; 0.4%). Two patients (0.4%) had missing data regarding implant system. No significant differences in antibiotic prophylaxis prescription occurred between the implant systems. Most surgeries (*n* = 353; 78.4%) were one-stage surgeries, followed by two-stage surgeries (*n* = 97; 21.6%).

### Antibiotic prophylaxis

Of the total study population of 450 patients, 205 (45.6%) received antibiotic prophylaxis. For straightforward implant placement, 96 (29.5%) received antibiotic prophylaxis, Table 1.

Procedures involving bone augmentation demonstrated markedly high antibiotic use, Table 2.

**Table 2:** Bone augmentation presented in six subgroups and antibiotic prophylaxis

Subgroups	Total	Antibiotics	
	*n*	Yes %	No %
Autologous bone	20	90.0	10.0
Synthetic bone	21	81.0	19.0
Autologous + synthetic bone	4	75.0	25.0
Autologous bone + membrane	3	100.0	0.0
Synthetic bone + membrane	17	94.1	5.9
Autologous + synthetic bone + membrane	5	100.0	0.0
**Total**	**70**	**88.6**	**11.4**

In the group of “Others” surgeries all patients received antibiotic prophylaxis, Table 1.

As Table 3 shows, the duration of prophylaxis varied significantly by type of surgery. Bone augmentation procedures showed a distinct shift toward longer antibiotic courses.

**Table 3:** Duration of antibiotic prophylaxis overviewed in patients not receiving any bone augmentation and the different bone augmentation subgroups (*n* = 450)

Type of surgery	No antibiotics %	Antibiotics 1 h preop %	3–10 days postop %	1 h preop + 3–10 days postop %
No bone augmentation	62.4	30.5	5.8	1.3
Autologous bone	10.0	40.0	15.0	35.0
Synthetic bone	19.0	66.7	9.5	4.8
Autologous bone + synthetic bone	25.0	25.0	0.0	50
Autologous bone + membrane	0.0	66.7	0.0	33.3
Synthetic bone + membrane	5.9	17.6	47.1	29.4
Autologous bone + synthetic bone + membrane	0.0	40.0	0.0	60.0
**Total**	**54.4**	**32.4**	**7.8**	**5.3**

### Predictors for antibiotic prophylaxis

The logistic regression analysis revealed significant predictors for antibiotic prophylaxis administration, as summarized in Table 4. Surgical complexity emerged as the primary determinant. In contrast, patient-specific factors, such as age, tobacco, and chronic diseases, showed limited associations.

**Table 4:** Logistic regression analysis of the outcome (antibiotic prophylaxis yes/no) and potential confounding variables (*n* = 404*)

Variable	Odds ratio	95% CI	*p* value
**Age (years)**			
20–44 45–59 60–74 75+	Ref 1.1 1.2 1.8	Ref 0.5–2.5 0.5–2.6 0.7–4.6	*0.739* *0.740* *0.234*
**Tobacco Use**			
No tobacco Cigarettes Snuff	Ref 1.2 0.3	Ref 0.4–3.1 0.1–1.1	*0.787* *0.067*
**Diabetes**			
**Cancer** **Osteoporosis**	1.8 1.9 ∞	0.6–4.7 0.6–6.4	*0.266* *0.307* *0.006*
**Type of surgery**			
Straight-forward Closed sinus lift without bone augmentation Immediate placement without bone augmentation Bone augmentation Closed sinus lift with bone augmentation Immediate placement with bone augmentation Others	Ref 17.5 4.1 14.7 68.6 10.8 ∞	Ref 6.7–45.9 0.6–26.4 5.3–40.3 8.8-537.1 1.7–101.0	*0.000* *0.133* *0.000* *0.000* *0.016* *0.000*
**Number of implants**			
Single implant 2–3 implants 4–6 implants	Ref 1.0 2.3	Ref 0.6–1.8 0.4–13.4	*0.964* *0.350*

^*404 of the 450 patient had complete data in all the potential counfounding variable fields and could hence be a part of the logistic regression analysis^

The likelihood ratio test demonstrated that incorporating surgical complexity variables significantly enhanced the predictive accuracy of the logistic regression model. This underscores the pivotal role of surgical complexity in determining the use of antibiotic prophylaxis in dental implant procedures. By comparing nested models, the likelihood ratio test assessed the impact of adding specific variables, allowing for a more precise evaluation of model fit. Given its sensitivity to changes in predictive performance, this statical approach effectively identified the significant influence of surgical complexity on antibiotic patterns. The results highlight that complex procedures, particularly those involving bone augmentation, were strong predictors of antibiotic use, whereas patient-specific factors played a lesser role.

## Discussion

This study addressed the following key questions: how frequently is antibiotic prophylaxis used in dental implant surgery in Sweden and what factors influence the decision to administrate antibiotic prophylaxis? By analysing real-world clinical data, the study showed the relationship between surgical complexity and antibiotic use. Less than half (45.6%) of the patients undergoing surgery received antibiotic prophylaxis. For straightforward surgeries, which comprised over 70% of all procedures, only 29.5% of the patients were prescribed antibiotics. These findings underscore a restrained approach to antibiotic use, contrasting with higher rates observed in other contexts [22–24, 28].

The low rate of antibiotic prophylaxis in straightforward surgeries aligns with recommendations from the 4th EAO Consensus Conference (2015) [13], which advises against routine antibiotics in uncomplicated cases. However, the results differ significantly from results reported in survey studies conducted in other countries. For instance, Arteagoitia et al. (2018) [22] reported that 85% of Spanish dentists prescribe antibiotics for implant surgeries, irrespective of complexity. Similarly, a study in Italy by Rodríguez Sánchez et al. (2019) [23] highlighted widespread prophylaxis use, unrelated to specific risk factors.

Unlike survey-based studies, the strength of this observational study lies in its reliance on real-world clinical data rather than self-reported habits, which are susceptible to recall bias and overestimation [29]. Survey studies evaluating the use of antibiotic prophylaxis in conjunction with dental implant surgery often lack procedural context, making it challenging to distinguish between straightforward and complex cases [22, 25]. This study’s detailed analysis of patient records allows for a nuanced understanding of the factors influencing antibiotic use, providing insights into actual clinical practices.

We chose the PDS Stockholm and Smile Dentistry for this study because they represent two major dental care providers in Sweden, offering a balanced perspective on both public and private dental healthcare. In 2020, over 4,150 dentists were employed in the public sector and more than 3,800 in the private sector, reflecting a significant presence of both sectors in Swedish dental care [30]. By including the PDS Stockholm and Smile Dentistry, we believe the study population may be representative of the general Swedish population, ensuring broader applicability of the findings. However, there is still a potential risk of selection bias that must be considered. By focusing on two major dental care providers, the study may inadvertently exclude certain groups of patients who seek care from smaller or regional providers. Additionally, the specific operational practices or patient demographics of PDS Stockholm and Smile Dentistry might not fully reflect those of other dental care providers in Sweden.

While this study provides valuable insights, several limitations must be acknowledged. The retrospective design limits the ability to establish causality between antibiotic use and surgical outcomes. Additionally, although the sample size was sufficient for primary analyses, sub-group analyses– particularly for rare surgical procedures or chronic diseases– may have been underpowered. The study also relied on pre-recorded data, which might not capture unquantifiable factors such as clinician perceptions of procedural complexity or patient-specific risks, like oral hygiene status or tobacco habits, that might have been taken in consideration by the clinician. Variability in surgical techniques across clinicians and centres, which could influence antibiotic decisions, was not comprehensively accounted for. The predominance of Straumann implants (91.7%) in this study reflects the implant system preferences commonly used in the participating clinics at the time of data collection. This may not necessarily reflect the boarder global market distribution of implant systems but is representative of the prevailing clinical practises within large Swedish public and private providers.

The medical morbidities observed in the study population were representative of the general Swedish population [31–33]. The significant association between complex surgical protocols, such as bone augmentation with membranes and increased antibiotic usage, suggests that clinicians perceive these procedures as having a higher risk of infection. For instance, procedures combining autologous and synthetic bone with membranes had the highest rates of antibiotic prophylaxis, with all patients receiving antibiotics in these categories. Such practices, while potentially reducing immediate postoperative infection risks, warrant scrutiny given the long-term consequences of antibiotic overuse [34]. The reliance on prolonged postoperative antibiotic regimens further complicates the balance between infection prevention and resistance management [35, 36].

Another critical observation was the variability in antibiotic regimens. Straightforward procedures predominantly involved single-dose prophylaxis or no prophylaxis at all, whereas bone augmentation surgeries frequently required extended courses. This finding resonates with the results presented in Salgado-Peralvo et al. (2021) [37], in which 49.8% of the participants of the survey study prescribed antibiotics both preoperatively and postoperatively for bone augmentation procedures. Such practices align with clinicians’ perceptions of higher infection risks but conflict with evidence from other surgical disciplines [38], which demonstrated that shorter antibiotic courses are often equally effective when surgical asepsis is maintained. This discrepancy underscores the need for evidence-based standardization of antibiotic clinical protocols in dental implant surgery. Prolonged and broad-spectrum antibiotic use not only risks fostering resistant bacterial strains but also exposes patients to unnecessary side effects, such as gastrointestinal disturbances and allergic reactions [34, 39].

To address these challenges, future research should focus on rigorous randomized controlled trials to evaluate the efficacy and necessity of antibiotics across various surgical scenarios, with a particular focus on implant placements with simultaneous bone augmentations of different kinds. Studies should investigate whether single-dose or short-course regimens suffice for complex procedures and explore strategies for integrating patient-specific risk factors into decision-making.

The findings of this study should be considered in light on ongoing global efforts to improve dental antibiotic stewardship. Despite dentists accounting for approximately 10% of all antibiotic prescriptions globally, research consistently shows high rates of unnecessary prescribing [40, 41]. A key challenge has been the lack of standardized outcome measures to evaluate stewardship interventions. Recently, an international consensus has led to the development of a core set (COS-DABS) designed to guide future research in dental antibiotic stewardship by promoting consistency in reporting and enabling meaningful cross-study comparisons [42]. Our study contributes real-world evidence supporting restrained antibiotic use in straightforward implant surgeries in Sweden, aligning with current stewardship principles and emphasizing the importance of surgical complexity as a key determinant for prophylactic prescribing. Integration of COS-DABS in future studies may help further refine guidelines and implementation strategies within this context.

## Conclusion

This study demonstrates that Swedish dentists largely follow a restrained approach to antibiotic prophylaxis in dental implant surgery, particularly in straightforward procedures. However, antibiotics were still prescribed in nearly one-third of such cases, highlighting the need to optimize antibiotic stewardship further and place greater emphasis on implementation efforts and the refinement of guidelines.

Given the high prophylactic antibiotic use in complex cases, further research is necessary to determine if shorter or more target regimens could be effective.

## Data Availability

All datasets used and/or analyzed during the current study are available from the corresponding author on reasonable request.
